# Collapse of the echinoid *Paracentrotus lividus* populations in the Eastern Mediterranean—result of climate change?

**DOI:** 10.1038/srep13479

**Published:** 2015-08-28

**Authors:** Erez Yeruham, Gil Rilov, Muki Shpigel, Avigdor Abelson

**Affiliations:** 1 Department of Zoology, Tel Aviv University, Ramat Aviv, Tel Aviv, 69978, Israel; 2 National Institute of Oceanography, Israel Oceanographic & Limnological Research, Tel-Shikmona, P.O.B. 8030, Haifa; 3 National Center for Mariculture, Israel Oceanographic & Limnological Research, P.O. Box 1212, Eilat 88112, Israel.

## Abstract

The European purple sea urchin (*Paracentrotus lividus*) is considered to be a key herbivore throughout its distribution range—North-East Atlantic and Mediterranean Sea. It was also abundant in its eastern distributional edge, on rocky habitats of the coastline of Israel, but its populations have recently collapsed, and today it is an extremely rare species in the region. Field and laboratory experiments, that were carried out in order to examine the impact of the recent sea surface temperature rise in the Eastern Mediterranean, showed massive urchin mortality when temperatures crossed 30.5 °C before reaching peak summer values. These results suggest that elevated seawater temperatures in recent years may be a main cause for the disappearance of *P. lividus* from the southeast Mediterranean Sea, which may indicate distributional range contraction in this region.

The increase in sea water temperature related to climate change is one of the major threats to marine biodiversity^1^,^2^. Changes in species distribution range and phenology traits, as well as mass mortality events have all been attributed to climate change^3^,^4^,^5^. A global average increase of 0.11 °C per decade was measured in the upper 75 meter layer during 1971–2010^6^, and this trend is expected to continue, as different models predict a further increase of 0.6–2 °C by the end of the 21st century^6^. The ecological effects of these temperature changes are not random, as some species are more strongly affected than others^7^,^8^.

Marginal seas like the Mediterranean may be especially susceptible to climate change impacts^9^. Previous assessments suggested that the Mediterranean Sea has indeed been highly affected by global warming through sea surface temperature (SST) increase of about 1 °C between the years 1974–2004^10^. This trend is expected to continue and water temperature has been projected to rise by up to 3.1 °C towards the end of this century^10^,^11^. Evidence from recent acute (~3 °C) but short warming periods (following exceptional heat waves) in the northwestern Mediterranean over the past 20 years demonstrated that high rise in temperatures often leads to mass mortality events. These events were not limited to local areas, as sessile organisms (mainly corals and sponges) along thousands of kilometers of coastline were affected^4^,^12^,^13^. The recent effects of water temperature rise are combined with multiple anthropogenic stressors, including over-exploitation, pollution and habitat destruction^14^,^15^.

A recently published time series from the offshore waters of Israel show an increase in SST of about 3 °C during the last three decades^16^. Here, we tested if a recent prominent decline of sea urchin populations, which are not commercially harvested on the Israeli coast, may be related to temperature changes. Within the Mediterranean, the Levant shores represent the eastern edge of distribution (trailing edge) of many native Mediterranean and Atlanto-Mediterranean species. It is also where sensitive native species might be expected to be gradually excluded by extreme conditions driven by climate change as this is already an extreme environment (highest temperature and salinity, and lowest primary productivity in the Mediterranean^17^). This may be the case for one of the ecologically-important species in the region, the sea urchin, *Paracentrotus lividus*, for which the SE Mediterranean is the warmest area in its distribution range. The SST in the southwestern distribution edge of *P. lividus*, the Canary Islands, is 5–6 °C lower during the summer months^18^, than along the Israeli coastline.

Past surveys (conducted in the 1970s) showed that *P. lividus* was very common on the Israeli coast, with densities of 2–10 individuals per m^2^ 19. In contrast, recent surveys show that *P. lividus* is extremely rare. In hundreds of dives that are part of very extensive benthic surveys of coastal rocky reefs between the years 2010–2014 in northern Israel (along 80 Km of coastline including inside an active marine reserve in Rosh Hanikra, and on reefs in Haifa Bay and the Carmel coast area), a total of 19 individuals were found^20^,^21^. Although there is no pre-collapse long-term density dataset, numerous discussions with fishermen, divers and researchers suggest that this species was still common until the early-2000’s, and its populations have collapsed over the past ca. 15 years. It is important to note that there are indications suggesting that *P. lividus* populations along the Lebanese and Syrian coastline are also declining^22^,^23^.

*P. lividus* is common in the northeast Atlantic and the Mediterranean Sea, mainly in shallow waters down to 20 meter depth. It is considered to be a key herbivore, because of its major role in structuring and controlling macroalgae assemblages, and thus in shaping the benthic seascape^24^,^25^,^26^. The decline of *P. lividus* populations observed today in the Levant is not limited to the Israeli coastline: Sukhn described a substantial deterioration of the population in southern Lebanon (Sidon) during the last decade^23^.

In this study, we tested the hypothesis that today’s very high summer SST along the Israeli coast (temperatures measured close to shore were between 30.5–31.5 °C during August both in 2011 and 2012 (Fig. 1), compared to about 29 °C in the early 1990s, may be beyond *P. lividus*’s thermo-tolerance limits and thus may have contributed to their population collapse.

## Materials and Methods

To test this hypothesis we conducted a series of field and lab experiments. Due to the present-day extreme rarity of the species in nature on the Israeli coast, the individuals used in our experiments were brought from the National Center of Mariculture (NCM), where they are reproduced from a Mediterranean original broodstock out of a 3-year old batch. The source of this broodstock was sea urchins collected from Mikhmoret (central Israel coast; the study site of this research). Reared urchins were exposed throughout their lifetime at NCM to water temperatures that varied between 21 °C in the winter to 29 °C in the summer. Prior to the experiments, the urchins underwent an acclimation to ambient Mediterranean Sea water for 2–4 months in an open water system on the Mediterranean coast.

The field and laboratory experiments were approved by Israel’s nature and parks authorities.

**Figure 1:**
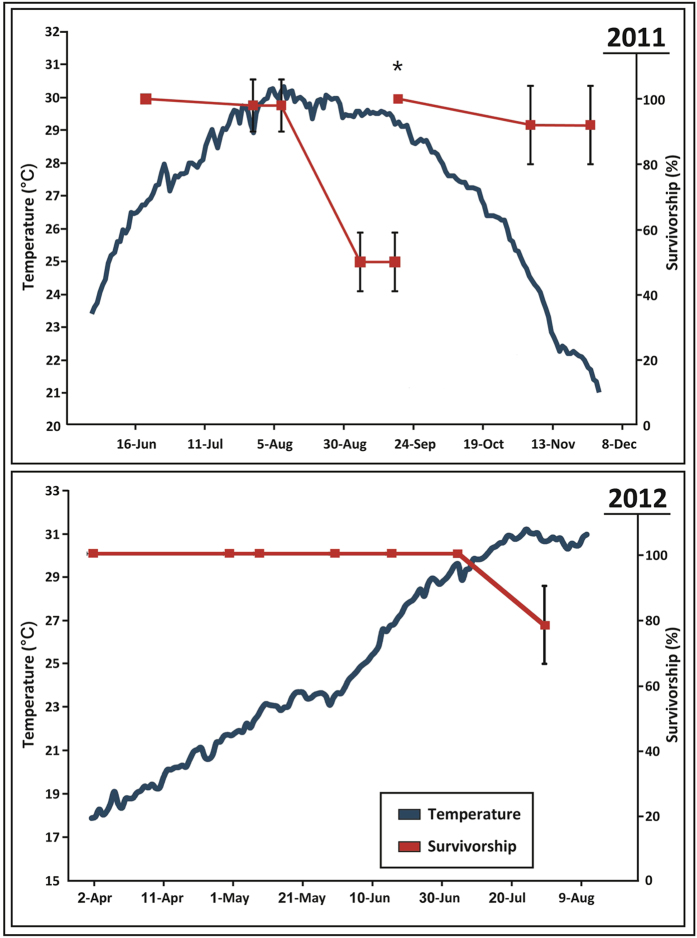
Seawater temperature and urchin survivorship in the field. Temperature (°C) and sea urchin average ± SE percent survival rate (N = 10, 5 urchins per repeat) during 2011 (upper chart) and 2012 (bottom chart). *The Survivorship increase in 2011 is due to restocking.

For the purpose of an inclusion/exclusion herbivore-effects experiment (as described elsewhere^27^), 50 adult *P. lividus* specimens were placed in 10 cages (50 × 50 × 20 cm, 5 urchins per cage) at a depth of 9 meters, 800 meters off Mikhmoret (central Israeli coast). The cages were fixed on a rocky reef, in an overgrazed area. The experiment was repeated twice, in July-November 2011 and in April-August 2012. Mortality rates were measured once a month by SCUBA diving. Due to the nature of the original experiment, in 2011, urchins were re-stocked in the treatment cages in early September after major mortality was detected in the August monthly assessments (hence the rise in “survival rate” in September, see Fig. 1).

### Laboratory experiment

The laboratory experiment was conducted during summer 2012, and it was specifically designed to differentiate between different possible effects of SST on urchin survival rate. In the facilities of Mevoot Yam Maritime School (Mikhmoret), 270 sea urchins were exposed to three temperature regimes (90 urchins in each), in a temperature-controlled system with running seawater (pumped-in directly from the sea). Total duration of the experiment was 94 days. The temperature treatments included: ambient seawater (i.e., seawater temperature following values in the open sea, temperature range during the experiment: 28.4–31.5 °C), ambient +2 °C (ambient seawater heated up by 2 degrees but following the seasonal trend, temperature range: 30.2–33.5 °C), and ambient –2 °C (ambient seawater cooled down by 2 degrees, temperature range: 27.1–29.9 °C). In the manipulated treatments (ambient +\−2 °C), prior to the experiment, sea urchins were acclimated to the altered water temperature at a rate of 1 °C/day. The urchins in each treatment were divided into five 24 L aquaria (18 individuals per aquarium). Each aquarium received separate water inflow, isolating the aquaria from each other to ensure no effects among aquaria. Survival rates were calculated per aquarium and then a mean was calculated for all aquaria per treatment. WSR (Weekly Survival Rate; the estimated proportion of animals alive on week w that is still alive on week w + 1) was calculated according to Van Der Toorn (2000) guidelines^28^.

### Statistical analysis

Data are presented as percentage, mean ± standard error (S.E.). The Kaplan-Meier survival analysis was performed, using SPSS 16.

## Results

Findings from both the field and laboratory experiments revealed a strong relationship between elevated water temperature during the summer months and *P. lividus* mortality. In both years, survival rate in the field cages decreased with the seasonal SST rise during the summer (Fig. 1, maximum temperature 30.5 °C in 2011, and 31.11 °C in 2012), and stopped almost entirely when SST dropped during the autumn (seen in the 1^st^ experimental period only). In the first experiment, survival rate was reduced to 50% by the end of August 2011, before restocking. In the second year, the survival rate reduced to 78% by the end of July 2012 (after temperature crossed the 30.5 °C threshold and just before the termination of the experiment—prior to peak summer water temperatures).

**Figure 2:**
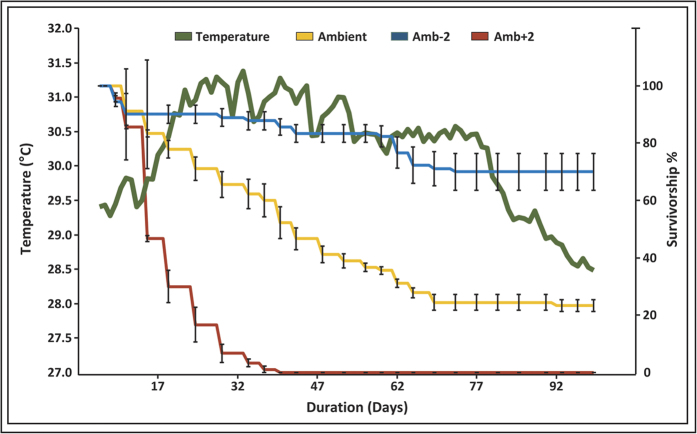
Effects of seawater temperature on urchin survivorship in the lab. Ambient water temperature (thick brown line) and sea urchin average ± SE percent survival rate (among five aquaria of 18 individuals per treatment) during the experiment (summer 2012), in the three treatments: AMBIENT – 2 °C (blue line), AMBIENT (green line) and AMBIENT + 2 °C (red line).

In the laboratory experiment, survival rate decreased sharply with the rise in water temperature, in the ambient and ambient +2 °C treatments (Fig. 2). In the heated treatment, all urchins died within three weeks. In the ambient treatment, mortality continued as long the temperature was above 30.5 °C (maximum water temperature was 31.5 °C) and ceased almost entirely when ambient temperature reduced below this value. Kaplan–Meier analysis revealed a significant difference (P < 0.0001) between the survival rates in the different temperature regimes. WSR was 0.97 in the ambient –2 °C treatment (final survival rate, 70 ± 6.5%), 0.89 in the ambient treatment (final survival rate, 22 ± 2.1%) and 0.63 in the ambient +2 treatment (final survival rate, 0).

## Discussion

The results of our field and laboratory experiments suggest that the recent, rapid SST rise may play a key role in the collapse of *P. lividus* populations along the Israeli Mediterranean coast. Even though this species is known to present strong fluctuations in population size^25^,^29^,^30^, we presume that this collapse is unprecedented because such a large scale, long-term (probably more than a decade) decline has not been recorded for this key species anywhere before. The urchins’ mortality in our study was not the result of a distinctive extreme weather event. During the summer of 2012, average daily seawater temperature was above 30 °C for 65 consecutive days including 12 days with temperature higher than 31 °C. The year of 2012 was not unique—the summer peak water temperatures during the last decade have often been higher than the observed lethal temperature of ~30.5 °C^31^. Over-harvesting, the main cause for *P. lividus* population decline elsewhere^26^,^32^, is not a factor along the Israeli coastline, since there is no commercial fishing of this species in the region, and poaching is rare. Other documented mortality factors such as predator hyper-abundance, toxic algae blooms, wave action and pollution^25^,^26^,^30^ have been ruled out as well, since no unusual episodes of these factors have been recorded. The possibility of exclusion by an invasive herbivorous fish, *Siganus rivulatus* and *Siganus luridus*, possible resource competitors of *P. lividus*^33^,^34^, was recently examined. Results from the field and laboratory experiments showed that reduced food availability can have sub-lethal impacts that may affect fitness in the long-term^27^. Therefore, temperature may be the proximal cause while competitive exclusion might play some role in the population collapse on the long-term; however, further work is required in order to determine its scope. Our results therefore provide strong evidence that the current SST temperature during the summer months is above the thermo-tolerance range of this species, and may have greatly contributed to the species near-extirpation in the region.

Disease is often considered as the driving mechanism behind sea urchin mortality due to elevated temperature^35^,^36^. A positive correlation between temperature rise and urchin mortality due to disease has been observed in the field, and in laboratory and mariculture systems^37^,^38^,^39^,^40^. Mass mortality of *P. lividus* due to bald sea urchin disease occurred in the Canary Islands during a particularly warm autumn in 2003^41^, but infection was also associated with unusually low wave height, and as a result, the mortality was limited to the intertidal populations and to that specific extreme event. Although we did not observe any external morbidity symptoms, such as spots, lesions, failure to hold spines erect and spine loss in the present work, we cannot rule out the possibility that temperature-induced disease may be the mechanism behind the discussed *P. lividus* population collapse. Deciphering this will require further study.

One may argue that the origin of the sea urchins (lab reared specimens) may affect their survival in the experimental setup during abrupt temperature rises due to the long-term rearing in relatively stable temperature conditions (with highest temperature of 29 °C). However, it may be similarly argued that the temperature range of the reared urchins served as a reliable simulation of the historical conditions experienced by the natural populations of *P. lividus* in the eastern Mediterranean in the past prior to the latest temperature increase during the last decades where it reached the lethal threshold of 30.5 °C in mid-summer.

We presume that the unexplained decline in population size in southern Lebanon during the previous decade^23^, which probably followed the decline observed along the Israeli coastline, is part of an expanding contraction of the urchins’ distribution from the hottest regions of the Mediterranean as a result of recent seawater temperature rise. We therefore predict that populations decline might soon occur farther northwest as the sea warms up in those areas as well.

## Additional Information

**How to cite this article**: Yeruham, E. *et al.* Collapse of the echinoid *Paracentrotus lividus* populations in the Eastern Mediterranean - result of climate change? *Sci. Rep.* **5**, 13479; doi: 10.1038/srep13479 (2015).
